# Spontaneous coronary artery dissection and atherosclerosis in a young man with systemic lupus erythematosus: A case report and literature review

**DOI:** 10.3389/fcvm.2022.951188

**Published:** 2022-08-10

**Authors:** Hongbo Huang, Xiaojuan Ma, Linjie Xu, Xin Wang, Dazhuo Shi, Fuhai Zhao, Ying Zhang

**Affiliations:** ^1^Graduate School of Beijing University of Chinese Medicine, Beijing, China; ^2^National Clinical Research Center for Chinese Medicine Cardiology, Xiyuan Hospital, China Academy of Chinese Medical Sciences, Beijing, China

**Keywords:** systemic lupus erythematosus, spontaneous coronary artery dissection, atherosclerosis, percutaneous coronary intervention, case report

## Abstract

**Background:**

Spontaneous coronary artery dissection (SCAD) is a rare coronary artery disease that frequently occurs in young, female patients without risk factors, and conservative treatment is often recommended for its management. The patient reported here is a male patient with systemic lupus erythematosus (SLE).

**Case summary:**

We described a 28-year-old man with SLE who presented with acute ST-segment elevation myocardial infarction (STEMI), and was diagnosed with SCAD through a long dissection of the left anterior descending branch (LAD) by coronary angiography. The patient was treated with percutaneous coronary intervention (PCI) with stent implantation. Ten years later, he developed in-stent stenosis and other coronary atherosclerosis and was retreated with PCIs. Based on this case and according to the literature review, the existing treatment and prognosis of SLE with spontaneous coronary artery dissection and atherosclerosis are discussed.

**Conclusion:**

Cardiovascular complications should be considered in patients with systemic lupus erythematosus, although they may not initially be atherosclerotic diseases. Attention should be paid to distinguish spontaneous coronary dissection in order to minimize missed or delayed diagnoses and take appropriate managements, as well as the development of atherosclerosis in SLE patients, and timely intervention has a better prognosis.

## Introduction

Spontaneous coronary artery dissection (SCAD) is defined as the formation of vascular false lumens due to a noninvasive and nonmedical separation of the coronary artery wall (1), with an incidence of 0.28 to 1.1% (2). Patients with SCAD usually present with acute coronary syndrome (ACS), and are misdiagnosed as atherosclerotic coronary artery disease whose management is different from SCAD (3). Therefore, current diagnosis and treatment are important. Its etiologies mainly include fibromuscular dysplasia (FMD), estrogen fluctuation periods such as pregnancy, connective tissue disease, and autoimmune diseases (4), such as systemic lupus erythematosus, which was the case in our patient here. SLE is an independent risk factor for cardiovascular disease (5), and cardiovascular disease has become the most common cause of death in SLE patients at late stage, especially atherosclerosis (6).

In our case, the young SLE patient presented with chest pain and was found a long dissection in the LAD by coronary angiography, so he was diagnosed with SCAD and underwent PCI. He survived well for years after PCI but still inevitably developed in-stent stenosis and other coronary atherosclerosis, and he received PCIs again.

## Case presentation

**Figure 1:**
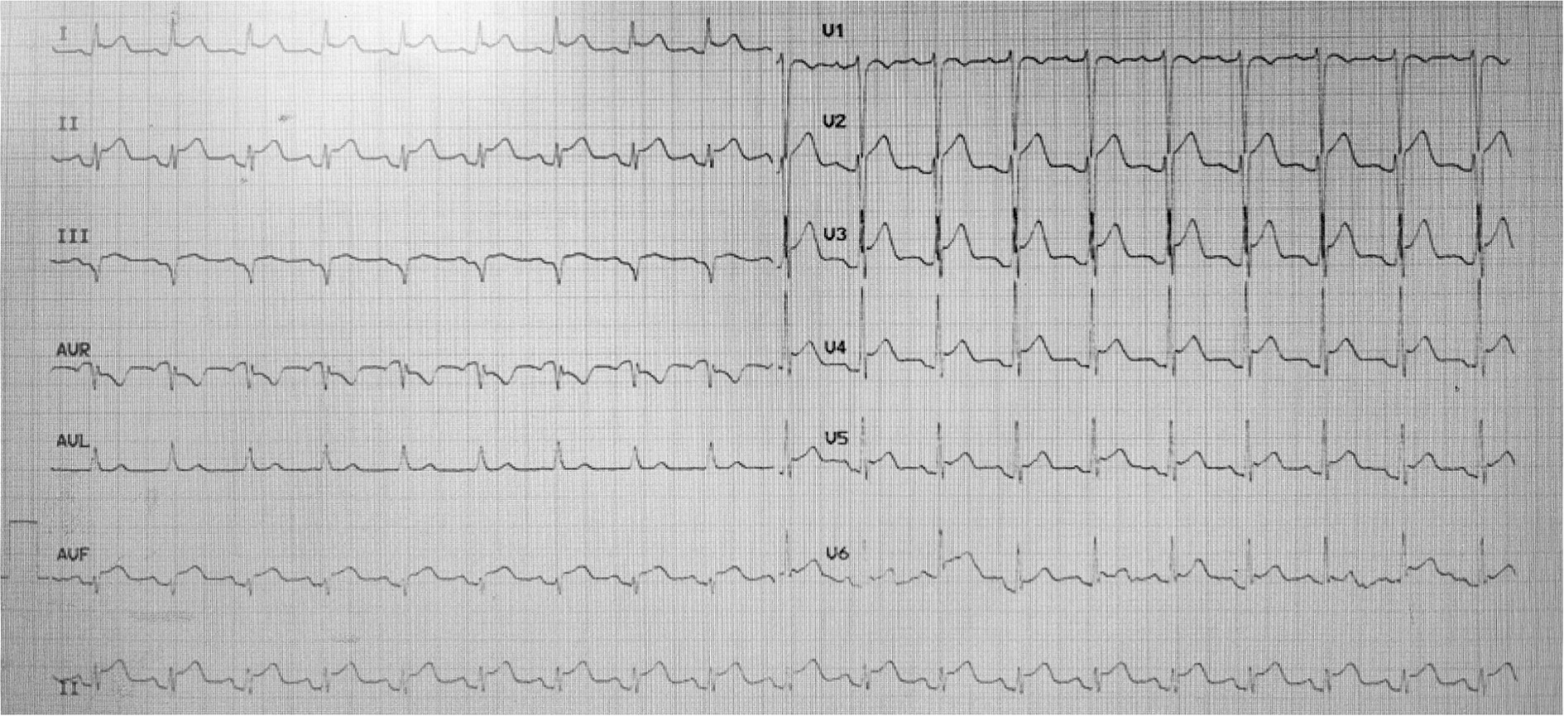
Emergency electrocardiogram in August 2011.

**Figure 2:**
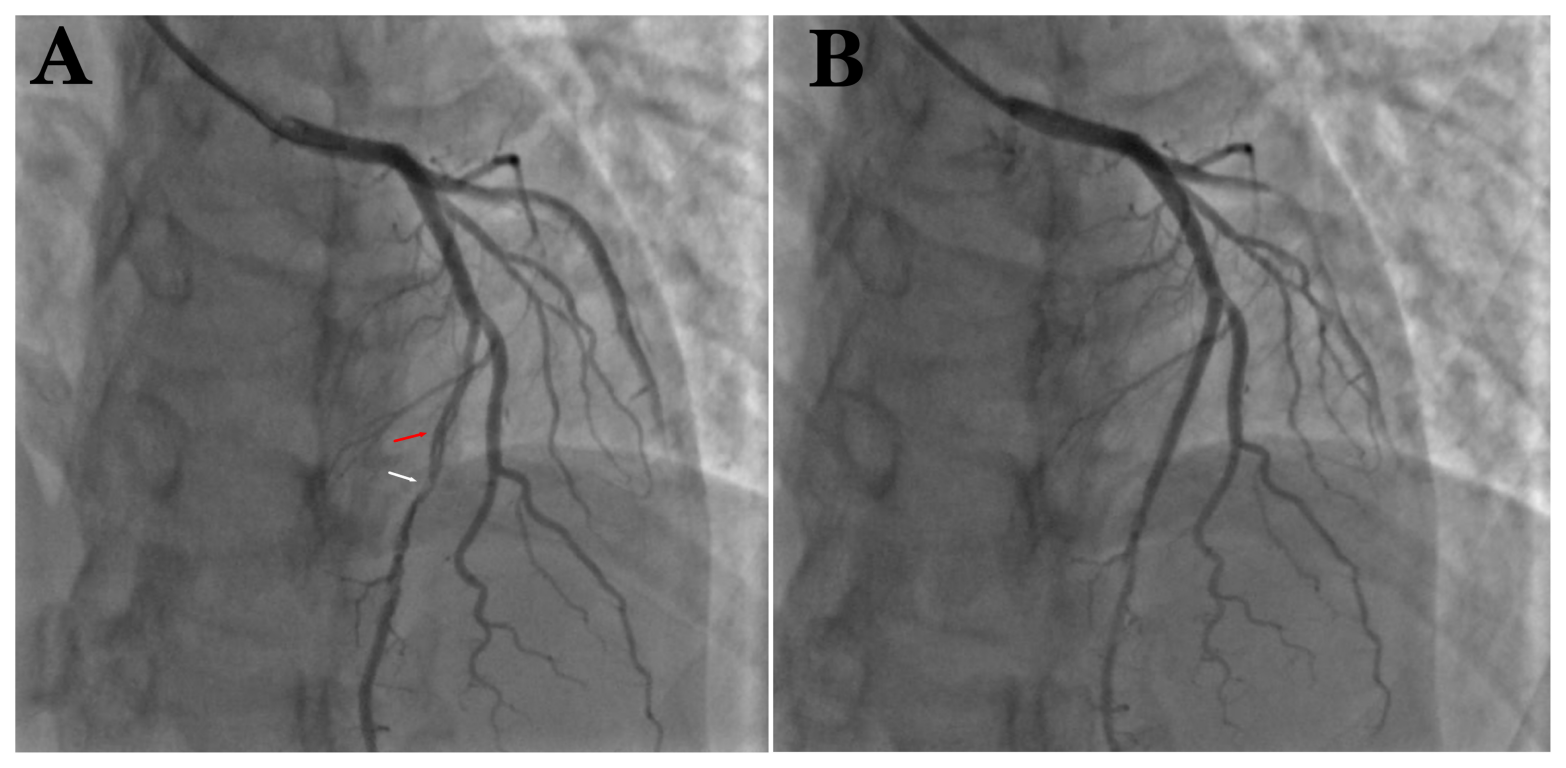
**(A)** Coronary angiography suggests spontaneous coronary artery entrapment (red arrow) and thrombotic shadow (white arrow) in LAD. **(B)** After implantation of 1 stent at the entrapment.

A young man was treated with prednisone in 2007 after a diagnosis of SLE due to the presence of malar rash and positive SLE-related antibodies, including antinuclear antibodies (ANA) (but negative anticardiolipin antibodies); however, the steroids were discontinued after the patient's symptoms had resolved. Since then, the patient has been hospitalized several times due to acute pericarditis, acute pleurisy, myocarditis, coronary arteritis, and lupus nephritis (LN), as well as repeated chest tightness and suffocation symptoms. In August 2011, at the age of 28 years, the patient developed persistent chest tightness and retrosternal pressure without obvious inducement, accompanied by profuse sweating, which could not be relieved spontaneously after rest, so he visited the emergency department of Xiyuan Hospital. An electrocardiogram showed ST-segment elevation in I, II, III, AVF, and V2–V6 (Figure 1), and cardiac enzyme levels were increased, so he was considered as STEMI. An emergency coronary angiography showed a long dissection and thrombus shadow since the diagonal branch in the left anterior descending branch (LAD) (Figure 2A), while the left main artery (LM), left circumflex branch (LCX), and right coronary artery (RCA) showed no abnormalities, and SCAD was diagnosed. To prevent further development of the coronary dissection in the anterior descending artery to the aorta, which could lead to disease aggravation, a stent (Lepu Medical Technology Co., Ltd. Beijing, China, LOT:201103001) was implanted at the LAD lesion (Figure 2B).

The patient had a long history of SLE, and newly discovered dyslipidemia and supraventricular tachycardia, with no history of smoking or alcohol consumption. After the PCI stent implantation, the patient had a multidisciplinary consultation and was given medical treatment, including aspirin 100 mg and clopidogrel 75 mg every day for antiplatelet therapy, atorvastatin 20 mg a day for lipid-lowering, metoprolol 12.5 mg twice daily for rate control, and methylprednisolone 50 mg per day for reducing the myocardial oxygen consumption. After discharge, the patient had taken dual antiplatelets for 1 year and clopidogrel for another year, but he had spontaneously stopped the glucocorticoid and statin due to concern of adverse reactions.

**Figure 3:**
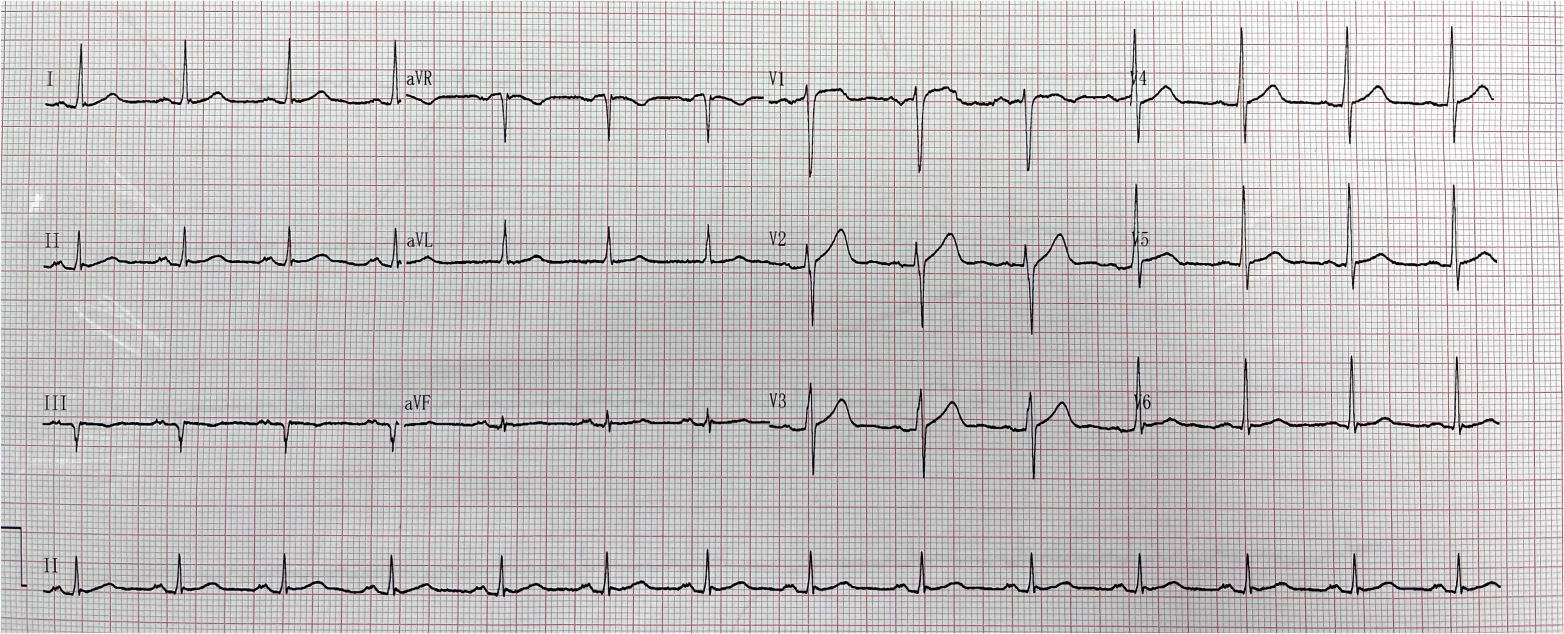
Emergency electrocardiogram in July 2021.

**Figure 4:**
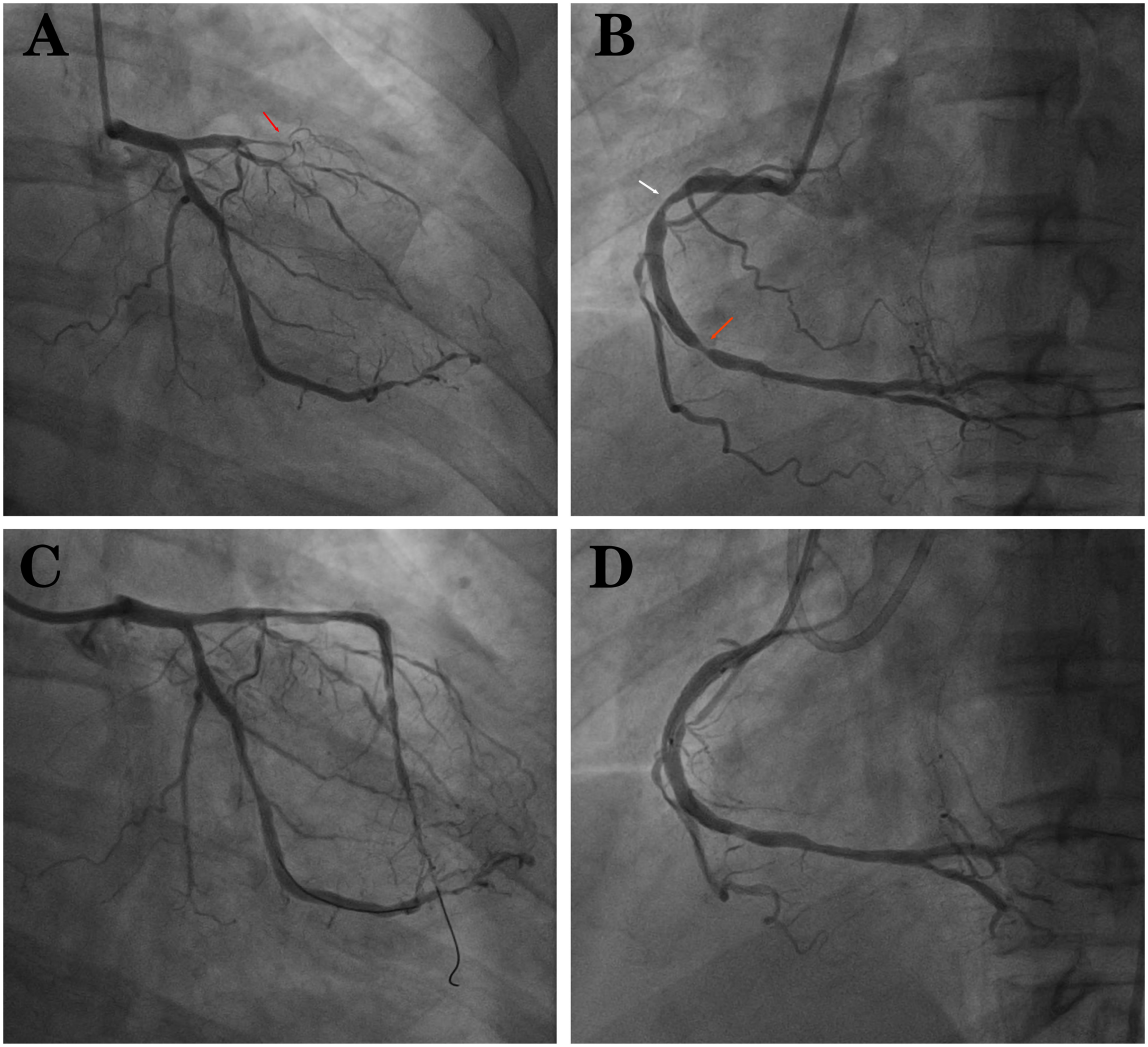
**(A)** LAD 100% occlusion (red arrow). **(B)** RCA with two stenoses (red and white arrows). **(C)** LAD after implantation of 3 drug balloons. **(D)** RCA after implantation of 1 stent and 1 drug balloon.

Years later (July 2021), the patient was readmitted to the hospital because he relapsed of chest tightness and suffocation which was worse than before. These symptoms occured during exercise and lasted for approximately 3–5 min and could be relieved after rest. Electrocardiogram returned to normal (Figure 3), and computed tomography angiography (CTA) that was performed on this admission suggested: (1) Severe stenosis at the proximal end of the LAD stent and moderate stenosis at the distal end of the stent. (2) Moderate stenosis at the proximal segment of the RCA; severe stenosis at the middle segment; and moderate stenosis at the distal segment. The patient was admitted for coronary arteriography, which showed there was 100% occlusion of the LAD from the stent and the collateral circulation was provided by the acute marginal artery (AM) (Figure 4A); there was 40% stenosis of the middle LCX; there was 80% localized stenosis in the middle RCA1 segment and 90% localized stenosis at RCA2 segment (Figure 4B). To open the occluded lesion of the LAD and the lesions of the RCA, PCI was performed. During PCI, because the guide wire could not correspond to the collateral circulation emitted by AM, the distal LAD was not successfully dredged. Therefore, the LAD operation was abandoned, the RCA lesions were treated first, and a drug-eluting stent (Essen Technology Co.,Ltd. Beijing, China, LOT:10200243) and a drug-coated balloon (Henan Qingzhou Medical Instrument Co., Ltd. Henan, China, LOT:06200611A1) were placed at the RCA, thus promoting the establishment of collateral circulation (Figure 4D). Three months later, the LAD lesion had retreated, and three drug-coated balloons (Henan Qingzhou Medical Instrument Co., Ltd. Henan, China, LOT:06210817A1, 06210621A1, 06210626A1) were placed (Figure 4C). The patient's chest tightness was relieved after the two PCIs, and the patient was given treatment for the secondary prevention of coronary heart diseases, with the main drug treatments being prednisone 15 mg a day, hydroxychloroquine 0.2 g twice daily and aspirin 100 mg per day, clopidogrel 75 mg per day, isosorbide mononitrate 20 mg every day, and rosuvastatin 10 mg once daily. Six months after discharge, the patient had no episodes of chest pain, and rheumatologists advised him to reduce the dose of prednisone and add azathioprine 125 mg per day for SLE. The main events of the patient showed in Table 1.

**Table 1:** Timeline of events.

**Timeline**	**Events**
11 August 2011	Persistent chest tightness and retrosternal pressure without obvious inducement, accompanied by profuse sweating. ECG showed ST-segment elevation in I, II, III, AVF, and V2–V6. Cardiac enzyme levels were increased. Emergency coronary angiography showed long dissection in left anterior descending branch (LAD). PCI was performed with one stent.
24 July 2021	chest tightness and suffocation, lasted for approximately 3–5 min and could be relieved after rest. ECG showed normal.
26 July 2021	CTA showed severe stenoses at the LAD stent and RCA.
27 July 2021	Coronary arteriography defined the locations of stenoses and a drug-eluting stent and a drug balloon were placed at the RCA.
19 October 2021	Three drug balloons were placed at LAD.

## Discussion

### Spontaneous coronary artery dissection

SCAD is an uncommon cause of myocardial infarction and sudden cardiac death, and it frequently occurs in young, female patients without cardiovascular disease risk factors (4). SCAD is a nonatherosclerotic disease (7). There are two mechanisms regarding the pathogenesis of SCAD. One is that the coronary intima is torn, which can occur due to various reasons, leading to bleeding in the media and the false lumen, which compresses the true lumen. The other is spontaneous rupture and bleeding of nutrient vessels of the arterial wall to form a hematoma, which compresses and causes stenosis (7). Patients with SCAD usually present with ACS, and studies have estimated that the incidence of SCAD is as high as 4% of patients with ACS (3), with chest pain being the most common symptom and the LAD being the most commonly involved vessel (8). SCAD in women occurs mainly in the postpartum period, while the main trigger in men is extreme physical activity (7).

With the development of medical imaging, the diagnosis of SCAD has increased than before. Despite some limitations, coronary angiography is still the first diagnostic tool for SCAD (9). Lesions are easily misdiagnosed as atherosclerotic plaques or coronary spasms when the imaging only shows luminal stenosis due to hematomas during SCAD. Familiarization with the angiographic variants of SCAD is the key to minimizing missed or delayed diagnoses (3). The other diagnostic techniques include intracoronary ultrasound, optical coherence tomography (OCT), and computed tomography coronary angiography (CTCA) to complement coronary angiography and confirm the diagnosis (3, 9).

The goal of treatment is to reduce the patient's symptoms and prevent recurrence (4). Conservative treatment is the mainstay of SCAD and most patients can heal completely over time (3). Pharmacological treatments, such as antiplatelet agents, β-blockers, angiotensin-converting enzyme inhibitors, and statins, are preferred when the patient's symptoms do not progress and hemodynamics are stable (3, 9). β-blockers have been shown advantages in reducing recurrent SCAD, but evidence is still lacking (4). Thrombolysis is contraindicated for SCAD because it may be effective to dissolve the thrombus in the false lumen (3). The dissolution of intraluminal thrombi may aggravate bleeding and worsen the dissection. Clinical deterioration after thrombolytic therapy in patient with SCAD has been described (10). PCI is selected when there is persistent ischemia, hemodynamic instability, and only single vessel dissection (2). However, PCI would fail due to the difficulty of technical operation or the development of intravascular hematoma displacement because of the stent placement, which even further leads to the spread of the hematoma (3, 9). Moreover, there is an increased risk of subsequent in-stent stenosis and thrombosis, and no studies have shown the duration of antiplatelet therapy in SCAD patients with PCI. In our case, the patient had received dual antiplatelet therapy after SCAD-PCI for 12 months and prolonged monotherapy. Previous studies have shown more options for CABG following failed PCI (11). Coronary artery bypass grafting (CABG) is used for left main and multivassel dissections (2), but graft occlusion is found to be more common in the postoperative follow-up (3). In addition, the use of statins seems to result in SCAD relapses (7). However, the use of statins in SLE patients and patients with dyslipidemia will still be considered, as shown later.

In retrospective studies, recurrent SCAD was found to mainly occur in female, myofiber dysplasia patients (7). The shifting in sex hormones in women during pregnancy, postpartum, perinatal and other periods may lead to connective tissue, hemodynamics and intravascular structural changes that weaken the vascular wall, resulting in intimal rupture or intramural hematoma formation and SCAD (12). Fibromuscular dysplasia (FMD) has been shown to affect the coronary arteries. The incidence of FMD in SCAD patients ranges from 31 to 72%, and this is important for the diagnosis and treatment of SCAD in a clinical screening of FMD (4). Angiography can show a peripheral arterial “beaded” pattern (13), and the possibility of SCAD should not be ignored when patients with FMD present with symptoms of chest pain. The patient in this case was male and had no FMD, but he used steroids because of SLE. In SLE patients, there appears to be an increased susceptibility to spontaneous dissection due to the chronic inflammation of the vessels (14). There was reported that steroid-induced SCAD (15), and steroid using was present in 0.66% of SCAD in a cohort study (16).

### SLE and atherosclerosis

For SLE patients, the development of glucocorticoid and immunosuppressive therapies targeting disease activity has led to a significant reduction in early mortality due to active lupus and infection, but the risk of death caused by cardiovascular disease among SLE paients has remained essentially unchanged (17). Meanwhile, SLE accelerates the development of cardiovascular diseases, especially atherosclerosis (6). The mechanism of atherosclerosis in SLE patients is complex and may interact under the conditions of traditional risk factors, lupus-associated factors, immune-inflammatory factors, and therapeutic factors (18).

#### Traditional cardiovascular risk factors

SLE patients have a high prevalence of traditional cardiovascular risk factors, such as dyslipidemia, hypertension, hyperglycemia, hyperhomocysteinemia, insulin resistance and other metabolic syndromes (19), and smoking, obesity, and sedentary lifestyle also accelerate the formation of atherosclerosis. Even after correcting for traditional cardiovascular risk factors, the prevalence of CVD in SLE patients has increased (20). The main risk factor is dyslipidemia in our patient, and he was hypertriglyceridemia that characterized by mild increases in triglycerides (TG) and decreases in high density lipoprotein-cholesterol (HDL-C), while low density lipoprotein-cholesterol (LDL-C) was borderline high. He did not belong to familial hyperlipidaemia. The combination of high TG and low HDL-C levels (together with the presence of small, dense LDL particles), referred to as atherogenic dyslipidaemia, is a common lipid disorder associated with increased cardiovascular disease risk (21). It has been shown that 48.1% of SLE patients treated with lipid-lowering drugs did not achieve the targeted lipid level (22). Dyslipidemia increases the risk of cardiovascular events in SLE patients, and dyslipidemia in SLE patients is also exacerbated by the disease activity (23). The mechanism of the interaction between them is complex and has not been fully elucidated. Normally, HDL has an antiatherosclerotic effect, mainly by allowing excess cholesterol to be excreted from the body. This cholesterol reversal mechanism allows the body to have cholesterol efflux capacity (CEC); however, CEC is impaired in lupus patients (24). In the inflammatory environment created by SLE, especially in the acute phase, HDL can be converted from inflammatory molecules to proinflammatory molecules that promote LDL oxidation, and Ox-LDL is further phagocytosed by macrophages to further form foam cells, which become the basis of atherosclerotic plaques (25). There is evidence that the systemic inflammatory burden in SLE patients disrupts cholesterol homeostasis (26), which contributes to dyslipidemia and exacerbates the formation of atherosclerosis in SLE patients.

#### Lupus-associated factors

In SLE, in addition to the direct vascular damage caused by inflammatory phenomena, immune complexes formed by auto-antibodies can also mediate endothelial cell damage (27), such as antinuclear antibodies (ANA), antiphospholipid antibodies (aPLs) and antidouble stranded DNA (anti-dsDNA) antibodies, among which anti-dsDNA antibodies are associated with abnormal activation of innate immune cells, leading to endothelial dysfunction and promoting atherosclerosis. Moreover, patients who are positive for anti-dsDNA antibodies are more likely to develop neutrophil extracellular traps (NETs) than negative patients (28). NETs are prominent fibrous networks of activated neutrophil membranes that themselves act as barriers to limit and eliminate pathogens at sites of inflammation; however, NETs degradation is blocked and prolonged in the autoimmune disease setting (29). NETs enhance immune stimulation, which damages the endothelium and accelerates the formation of atherosclerosis (30). Some cytokines that will be overexpressed in SLE, such as IFN-α (31), INF-γ (32) and TNF-α(33) lead to inflammatory cell recruitment, stimulate macrophage activation, induce matrix metalloproteinase secretion, and upregulate adhesion molecule expression to promote atherosclerosis.

#### Treatment-related factors

As the disease progresses, the therapy of SLE can also lead to the development of atherosclerosis (34). Glucocorticoids, as basic drugs, play an irreplaceable role in the treatment of acute SLE and vital organ damage and have been instrumental in reducing mortality in the active phase of SLE in recent years (35). However, the long-term use of steroids will cause continuous high levels of glucocorticoids in the body, which increases the risk of concurrent cardiovascular events in SLE patients (36). The increased prevalence of traditional cardiovascular risk factors may also be related to the development of hyperlipidemia, hypertension and obesity induced by glucocorticoids (37). During long-term maintenance therapy, the use of glucocorticoids should be minimized and discontinued if possible (34). Ruiz-Arruza et al. (38) showed that reducing the dose of oral prednisone, combined with other treatments such as immunosuppressive or biologic drugs, can reduce glucocorticoid-related damage, thereby improving cardiovascular outcomes. In a cohort study in China, the use of hydroxychloroquine and azathioprine in SLE patients increased the probability of survival (39). Hydroxychloroquine (HCQ), as an antimalarial drug, not only has a good effect on SLE disease activity and prevention of injury but also has a significant effect on lowering traditional cardiovascular risk factors such as dyslipidemia and diabetes. In fact, antimalarial therapy has been regarded as a potential atheroprotective agent (40). HCQ may play a role in lowering cholesterol levels by upregulating LDL receptors, potentially counteracting the negative effects of prednisolone on blood lipids and slowing the development of atherosclerosis (41, 42). In addition, HCQ can also always reduce the risk of thrombosis by inhibiting platelet aggregation (43). Therefore, all SLE patients should be treated with HCQ, as long as there are no contraindications (44). However, attention should be given to the development of hydroxychloroquine maculopathy, and the patients should have regular eye screenings (44). Immunosuppressive drugs such as methotrexate (MTX) and azathioprine (AZA) should be used when GC in combination with HCQ has poor efficacy (5). Biological therapy is mainly used in the clinical situation in which SLE patients remain resistant to conventional immunosuppressive agents, but for all disease manifestations, it is difficult to solve all of the problems with only one biological therapy (17, 34).

For the risk of cardiovascular complications in SLE patients, the commonly used Framingham risk score (FRS) underestimates the cardiovascular risk of SLE patients. A retrospective study found that the modified FRS using 2.0 multiplier has increased the sensitivity of this indicator from 0.13 to 0.31 (45). Although the 2019 European League Against Rheumatism (EULAR) guidelines recommend the application of SCORE to assess the risk of cardiovascular disease in patients for 10 years, the risk in SLE patients is still underrated (5). At present, there is no direct comparison of the performance of most commonly used general risk assessment tools in SLE. Therefore, it is recommended to conduct a comprehensive assessment of traditional and disease-related risk factors, and to provide individualized prevention and treatment according to the patient's situation (46).

#### Clinical strategies for SLE with atherosclerosis

In the prevention and treatment of atherosclerosis in SLE patients, the first is the control of risk factors, including but not limited to smoking cessation, maintaining an ideal weight, avoiding a sedentary lifestyle, and controlling blood pressure, blood glucose, blood lipids, and homocysteine (20). Dyslipidemia should be treated aggressively. Statins in SLE patients are still controversial, and long-term use of statins may be associated with drug-induced lupus (47, 48). However, it has also been shown that statins reduce the premature mortality of patients with autoimmune rheumatic diseases (49). Watanabe et al. (50) showed that starting statins within 3 months of the onset of SLE reduced the risk of thrombosis. Statins should still be considered based on the patient's lipid levels and the presence of other risk factors (39). In SLE patients, their blood pressure should be more strictly controlled compared to the general population. Thiazide diuretics should be used with caution in SLE (51). Folic acid can reduce homocysteine serum concentrations and reduce its toxicity to the endothelium and can be used as a preventive treatment (21). Vitamin D reduces endothelial damage by reducing NETosis activity and may also be a targeted therapy for SLE (29).

In SLE patients with preexisting atherosclerosis, cardiovascular drugs are necessary. Auto-antibodies have procoagulant activity, and low-dose aspirin can reduce the risk of vascular thrombosis prophylactically (52). Patients with SLE may be more prone to adverse cardiac outcomes after coronary revascularization by PCI or CABG, so the risk reduction after revascularization should be aggressive (53). Cohort studies in Taiwan have also demonstrated that SLE patients require repeat PCI within 1 year compared with non-SLE patients (54).

## Conclusion

In summary, we reported a case of systemic lupus erythematosus with spontaneous coronary dissection and coronary atherosclerosis in a young man. When dealing with patients with SLE, attention should be given to cardiovascular complications, although it may not start out as atherosclerotic disease. Although SCAD is rare, it has a high risk level as an attack, so clinicians should take note of the young patients with chest pain without cardiovascular diseases previously and respond proportionally. At the same time, attention should also be paid to the development of atherosclerosis in SLE patients.Traditional cardiovascular risk factors and a combination of medications are used to reduce the damage caused by glucocorticoids, to prolong the survival time of SLE patients and improve the quality of life.

## Data availability statement

The original contributions presented in the study are included in the article/supplementary material, further inquiries can be directed to the corresponding author/s.

## Ethics statement

Written informed consent was obtained from the individual for the publication of this case report.Written informed consent was obtained from the individual for the publication of any potentially identifiable images or data included in this article.

## Author contributions

HH, XM, LX, and XW organized data and figures, performed the literature research and wrote the manuscript. FZ performed PCIs and provided figures. YZ and DS provided study concept and critical revision of the manuscript for intellectual content. All authors contributed to the manuscript production and the final revision.

## Funding

YZ received funding from the Fundamental Research Funds for the Central Public Welfare Research Institutes of China (Grant No. ZZ13-YQ-008) and the key project of Science and Technology Innovation Project of China Academy of Chinese Medical Sciences (Grant No. CI2021A03115).

## Conflict of interest

The authors declare that the research was conducted in the absence of any commercial or financial relationships that could be construed as a potential conflict of interest. The reviewer JC has shared affiliation with some of the authors, HH and LX, to the handling editor at time of review.

## Publisher's note

All claims expressed in this article are solely those of the authors and do not necessarily represent those of their affiliated organizations, or those of the publisher, the editors and the reviewers. Any product that may be evaluated in this article.
